# Interactions of alkali cations with glutamate transporters

**DOI:** 10.1098/rstb.2008.0246

**Published:** 2008-10-31

**Authors:** David C. Holley, Michael P. Kavanaugh

**Affiliations:** Center for Structural and Functional Neuroscience, University of MontanaMissoula, MT 59812, USA

**Keywords:** glutamate transporter, chloride channel, ion binding, synaptic transmission

## Abstract

The transport of glutamate is coupled to the co-transport of three Na^+^ ions and the countertransport of one K^+^ ion. In addition to this carrier-type exchange behaviour, glutamate transporters also behave as chloride channels. The chloride channel activity is strongly influenced by the cations that are involved in coupled flux, making glutamate transporters representative of the ambiguous interface between carriers and channels. In this paper, we review the interaction of alkali cations with glutamate transporters in terms of these diverse functions. We also present a model derived from electrostatic mapping of the predicted cation-binding sites in the X-ray crystal structure of the *Pyrococcus horikoshii* transporter Glt_Ph_ and in its human glutamate transporter homologue EAAT3. Two predicted Na^+^-binding sites were found to overlap precisely with the Tl^+^ densities observed in the aspartate-bound complex. A novel third site predicted to favourably bind Na^+^ (but not Tl^+^) is formed by interaction with the substrate and the occluding HP2 loop. A fourth predicted site in the apo state exhibits selectivity for K^+^ over both Na^+^ and Tl^+^. Notably, this K^+^ site partially overlaps the glutamate-binding site, and their binding is mutually exclusive. These results are consistent with kinetic and structural data and suggest a plausible mechanism for the flux coupling of glutamate with Na^+^ and K^+^ ions.

## 1. Introduction

In order to maintain chemical neurotransmission, prompt removal of neurotransmitter following synaptic release must occur. This is necessary for terminating the actions of the transmitter on the postsynaptic cell and for maintaining specificity of synaptic communication. In the case of glutamate, the principal excitatory neurotransmitter in the nervous system of vertebrates, it is also necessary to control extracellular transmitter levels in order to prevent excitotoxic damage from excessive receptor activity. This task is chiefly accomplished through reuptake mediated by excitatory amino acid transporters (EAAT1–5; SLC1A1–5) present in the plasma membranes of glia and neurons throughout the nervous system (Tzingounis & Wadiche 2007). A hallmark of glutamate uptake is co-transport with Na^+^ and H^+^ and countertransport of K^+^, indicative of a mixed co-transport–exchange carrier mechanism (Kanner & Sharon 1978). Another hallmark of the transporters is an intrinsic chloride conductance that is generally increased during transport in cells expressing exogenous or endogenous transporters (Fairman *et al*. 1995; Picaud *et al*. 1995; Wadiche *et al*. 1995; Billups *et al*. 1996; Wadiche & Kavanaugh 1998). A number of studies have addressed questions about the detailed mechanism of glutamate uptake and chloride channel gating, but questions still remain about fundamental features such as the binding order of transported solutes and the location of binding sites and permeation pathways (for reviews see Danbolt (2001), Grewer & Rauen (2005) and Tzingounis & Wadiche (2007)).

**Figure 1:**
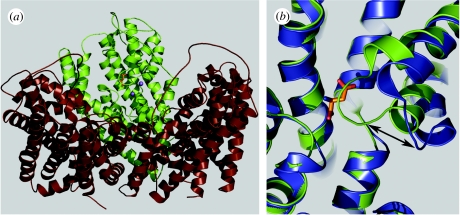
Glt_Ph_ structure model based on Yernool *et al*. (2004). (*a*) Three subunits form a bowl that penetrates the plasma membrane. One of the subunits is depicted with aspartate and two bound Tl^+^ (Boudker *et al*. 2007). (*b*) Single subunit overlays of the occluded (green) and open (blue) conformations with aspartate bound to the occluded form. The arrow highlights the HP2-loop conformational change between the open and occluded forms.

The solution of the crystal structure of Glt_Ph_, a glutamate transporter homologue from *Pyrococcus horikoshii*, represents a critical advance towards a better understanding of the transport mechanism. The transporter has a trimeric architecture (Yernool *et al*. 2004). Each of the three subunits has eight transmembrane domains with two re-entrant helical hairpin loops (HP1 and HP2) that dip into the membrane from opposite sides (figure 1). Structural evidence suggests that each subunit in the trimer binds its amino acid substrate independently of the others (Yernool *et al*. 2004; Boudker *et al*. 2007). Functional evidence also supports the idea that each subunit operates as both a self-contained glutamate transporter and chloride channel (Grewer *et al*. 2005; Koch *et al*. 2007; Leary *et al*. 2007).

## 2. Computational methods

Human EAAT3 sequence (GenBank; http://www.ncbi.nlm.nih.gov) and the EAAT3 R447C mutant sequence were aligned with the Protein Data Bank (PDB) sequences for the Glt_Ph_ homologue (2NWX and 2NWW; Yernool *et al*. 2004; Boudker *et al*. 2007). Homology models were built by threading the aligned EAAT3 sequences along their respective PDB coordinates using the SwissProt server (http://swissmodel.expasy.org//SWISS-MODEL.html). The resulting models were optimized through local energy minimizations of regions with steric and electrostatic interference using the Amber7 force field in the Tripos SYBYL7.3 platform. Valence shell mapping and calculations were performed using the Fortran program Vale (courtesy of Enrico Di Cera and Thierry Rose; Washington University, St Louis, MO) using a 3.4 Å probe radius and a grid size of 0.1 Å (Nayal & Di Cera 1994) on Glt_Ph_ and homology model PDB files. Only coordination sites within a 10 Å radius of the substrate α-carbon imported from the aspartate-bound crystal structure were considered as candidates for the Na^+^- and K^+^-binding sites. Calculations for each cation–oxygen pair are given by *ν*=(*R*/*R*_1_)^−*N*^, where *R* is the distance of an oxygen atom to the cation; *R*_1_ is the distance given valence=1.0; and *N* is an empirically derived exponent specific to each cation (Nayal & Di Cera 1994, 1996; Page & Di Cera 2006). The values for *R*_1_ and *N* were determined through the analysis of metal-oxide crystal structures and define an estimated ideal bond strength as *ν*=1.0. Although all pertinent regions of the 10 Å binding arena are likely to be water accessible, no water molecules were included in the valence mapping in order to reduce noise (Nayal & Di Cera 1994). Consistent with recent studies, we chose a valence cut-off of *ν*=0.9 for likely candidate-binding sites (Ogawa & Toyoshima 2002; Rakowski & Sagar 2003; Page & Di Cera 2006). The three-dimensional coordinates of valence maps that contain multiple, clustered valence sites within a 2 Å radius were included as a single site and we reported the highest estimated valance within each site. l-Glutamate was docked using Gold (http://www.ccdc.cam.ac.uk/) into the EAAT3 and Glt_Ph_ models either containing Na^+^ or K^+^, as determined by valence mapping, or containing no cations. The top-ranked structures determined by ChemScore (http://www.ccdc.cam.ac.uk/) were incorporated into the appropriate models and average rankings from three separate Gold docking runs were reported. In order to evaluate estimations of absolute affinity of substrates in different models, Δ*G* scores were extracted form ChemScore and reported as an average over the three docking runs. In order to optimize models, iterative valence mapping and docking runs were performed.

## 3. Thermodynamic coupling of glutamate and cation fluxes

Influx of glutamate, Na^+^ and H^+^, and efflux of K^+^, during a transport cycle results in a net flow of positive charge into the cell that can be recorded with a voltage clamp circuit. A tight stoichiometric coupling is inferred from the effects of these cations' concentration gradients on the reversal potential of the pharmacologically isolated transport current. The transporter reversal potential (the equilibrium membrane potential at which there is no net transport) follows the predictions of the free energy equation for the coupled transport of one glutamate molecule with one proton and three Na^+^ ions and countertransport of one K^+^ ion during each uptake cycle (Zerangue & Kavanaugh 1996*a*; Levy *et al*. 1998). An overarching question concerns the mechanism of this tight coupling. The simplest type of theoretical kinetic scheme consistent with the data is an alternating access carrier model (Jardetzky 1966). The precise binding order of ions and glutamate is unresolved; the kinetic data suggest that one or two of the three Na^+^ ions bind before glutamate (Tzingounis & Wadiche 2007). The solution of two crystallized structural states of Glt_Ph_ has provided critical new information and suggests a possible structural mechanism for an alternating access transport scheme (Boudker *et al*. 2007). The trimer subunits contain eight transmembrane domains (TM1–8) and two α-helical, re-entrant hairpin loops (internal HP1 and external HP2). Gouaux and colleagues have suggested that these loops could function as gates, allowing alternating substrate access. In the aspartate-bound state, aspartate is occluded between the tips of the two re-entrant loops, and charge pairing between the bound aspartate and residues including a conserved arginine in TM8 of the transporter stabilizes the complex. Two anomalous densities can be resolved that are associated with the replacement of Na^+^ by Tl^+^ in the aspartate complex. By contrast, in crystals produced from transporter complexed with the bulky non-transported inhibitor d,l-threo-β-benzoylaspartic acid (TBOA), the external HP2 loop is swung outwards, approximately 10 Å, and one of the ion densities is disrupted. These data suggest a possible docking trajectory and structural gating mechanism for the first hemicycle of an alternating access kinetic scheme (figure 1). A presumed third state, which would allow the substrate and the cations access to the cytoplasm, has thus far eluded crystallization.

## 4. Thermodynamically uncoupled chloride flux

In addition to the flux of stoichiometrically coupled ions, chloride flux also occurs through the transporters (for a review, see Tzingounis & Wadiche (2007). This was first suggested by the presence of a chloride current associated with the activation of the native glutamate transporters in retinal neurons and glia (Eliasof & Werblin 1993; Picaud *et al*. 1995; Billups *et al*. 1996; Eliasof & Jahr 1996), and it has also been demonstrated to occur with EAATs exogenously expressed in different cell systems (Fairman *et al*. 1995). The channel is also found in neutral amino acid transporter members of the eukaryotic SLC1 family (Zerangue & Kavanaugh 1996*b*). At some synapses, the chloride conductance appears to play feedback roles in synaptic signalling, which are entirely distinct from the effects of uptake on glutamate dynamics (Veruki *et al*. 2006; Wersinger *et al*. 2006). The chloride channel itself seems to be intrinsic to the transporter, as the mutation of specific residues leads to discrete changes in anion channel properties (Ryan *et al*. 2004; Huang & Vandenberg 2007). The chloride channel function is also conserved in a reconstituted bacterial homologue, further supporting the idea of a channel in the transporter structure (Ryan & Mindell 2007). The net current activated by glutamate (reflecting both the stoichiometrically coupled and Cl^−^ currents) has a distinct reversal potential in each EAAT isoform, suggesting that each has a fixed and unique Cl^−^ current magnitude relative to the stoichiometrically coupled current. The relative magnitude of anion conductance : coupled current follows the sequence EAAT4∼EAAT5>EAAT1>EAAT3>EAAT2. In each transporter, the anion conductance displays a chaotropic selectivity sequence SCN^−^>${\text{ClO}}_{4}^{-}$>${\text{NO}}_{3}^{-}$>I^−^>Cl^−^>F^−^≫gluconate^−^. SCN^−^ is approximately 70-fold more permeant than Cl^−^ (Wadiche & Kavanaugh 1998). The glutamate-dependent anion conductance is strongly affected by the identity of the alkali cation co-transported with glutamate; Li^+^ can substitute for Na^+^ in some isoforms to support glutamate transport but is much less efficacious at activating the anion conductance (Borre & Kanner 2001). With Na^+^ present, there is a tonic anion conductance in the absence of glutamate (Bergles & Jahr 1997; Tao *et al*. 2006).

Replacement of Cl^−^ with impermeant anions such as gluconate does not affect the transport of glutamate (Wadiche *et al*. 1995). Thus, Cl^−^ flux may be considered thermodynamically uncoupled from glutamate flux, and seems to involve a channel-like mechanism instead. Indeed, glutamate-dependent anion current fluctuations have been observed with endogenous and exogenous transporter expressions that are consistent with a stochastically gated channel that is kinetically related to the glutamate transport cycle (Picaud *et al*. 1995; Wadiche & Kavanaugh 1998). Kinetic analysis of the glutamate concentration dependence of transport and chloride conductance suggests that each subunit in the trimer harbours both a chloride channel and a glutamate transporter, and each subunit functions independently (Koch *et al*. 2007; Leary *et al*. 2007). Kinetic models may be able to unify the distinct channel and transport functions by representing a subset of the Markov states in the transport cycle as open-channel states (Grewer *et al*. 2000; Otis & Kavanaugh 2000; Bergles *et al*. 2002).

## 5. Structural model for flux coupling

An initial picture of the interaction of alkali cations with glutamate transporters has emerged from anomalous difference maps seen in Tl^+^-soaked Glt_Ph_ crystals (Boudker *et al*. 2007). Two densities were seen that were selectively diminished by Na^+^. One of these (site 2) was not seen in transporters complexed with TBOA, which also caused a large outward displacement of the HP2 loop (figure 1). Because the crystal diffraction resolution was insufficient to localize Na^+^ ions interacting with Glt_Ph_, and owing to the possibility that the Tl^+^ sites might not accurately represent these sites, we used the electrostatic mapping algorithm Vale (Nayal & Di Cera 1994) to examine potential Na^+^-, K^+^- and Tl^+^-binding sites in the transporter. Electrostatic calculations with this algorithm have been successfully used to predict Na^+^- and K^+^-binding sites in proteins including the Na^+^, K^+^-ATPase (Nayal & Di Cera 1996; Ogawa & Toyoshima 2002; Rakowski & Sagar 2003).

**Figure 2:**
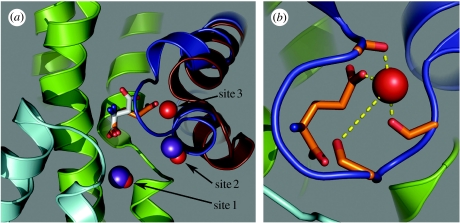
Identification of a novel sodium site in EAAT3. (*a*) Sodium sites 1–3 (red spheres) are depicted, showing the proximity of sites 1 and 3 to the crystal structure thallium sites (purple spheres) identified by Boudker *et al*. (2007). The site 2-bound ion interacts with the HP2 helix dipole and the γ-carboxyl group of docked glutamate (orange sticks). Overlaid with the docked glutamate molecule is the aspartate (white sticks) that was resolved in the crystal structure. The HP1 loop is depicted in cyan, with the occluding HP2 loop in dark blue. (*b*) Na^+^ docked at site 3 is coordinated by α-carboxyl groups in the HP2 loop and by the γ-carboxylate of bound glutamate.

The Glt_Ph_ and EAAT3 models used for the valence calculations consist of four basic structures: the two loop conformers (HP1 open or closed) with or without the bound amino acid substrate (see figure 1 and §2). A total of four binding sites were identified corresponding to potential ion coordination sites above the 0.9 valence cut-off within a 10 Å radius of the substrate-binding site. In several of these ion-binding sites, the valence was dependent on the loop position and the presence or absence of glutamate or aspartate (table 1). For both the Glt_Ph_ and the EAAT3 models, two sites (sites 1 and 2) are predicted, which correspond very well (less than 1.4 Å) to the Tl^+^ densities resolved in the occluded Glt_Ph_ crystal structure determined by Boudker *et al*. (figure 2; table 1). The valence of site 1 is relatively independent of substrate binding and loop conformation, and is therefore likely to coordinate a sodium ion before glutamate is bound. By contrast, site 2 is coordinated by dipoles formed from α-helices in the closed HP2 loop and TM7. This is consistent with the crystal structures, where one Tl^+^ density (site 1) was unaffected by the HP2-loop state, but the second density (site 2) was lost in the TBOA-bound open-loop state (Boudker *et al*. 2007).

**Figure 3:**
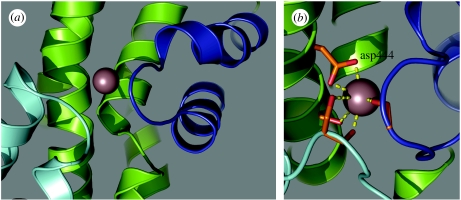
Interactions of K^+^ at site 4. (*a*) Site 4 overlaps with the aspartate-binding site in the crystal structure of the aspartate-bound archaeal homologue. (*b*) Bound K^+^ is predicted to interact directly with D444, a residue also involved in glutamate binding (Teichman & Kanner 2007).

Electrostatic mapping of EAAT3 also revealed a coordination shell for a novel site (site 3, figure 2) whose interaction with Na^+^ was favoured by bound glutamate. Na^+^ is predicted to be more stably coordinated in the occluded, substrate-bound state (*ν*=0.91) than in either the open or closed apo states (*ν*=0.72 or 0.62, respectively; table 1). This glutamate effect arises from the contribution of a ligating oxygen to the site 3 coordination shell by the γ-carboxyl group of bound glutamate. Steric and van der Waals constraints between the bound ion and the occluding HP2 loop may also confer sodium selectivity at site 3, which shows poor predicted K^+^ and Tl^+^ valences (*ν*<0.3). Site 3 thus represents a potential third Na^+^-selective binding site that could participate in cooperative binding of glutamate (figure 2). Valence modelling of Glt_Ph_ predicts a tenuous coordination for Na^+^ in site 3 (*ν*=0.61), but does not predict a viable coordination site for Tl^+^ at this site (*ν*<0.3), which is consistent with the absence of Tl^+^ density at site 3 in Glt_Ph_. Electrostatic mapping also predicts a fourth cation-binding site in the transporter (figure 3). Site 4 exhibits a marked selectivity for K^+^ over Na^+^ (table 1; figure 3). This site also substantially overlaps the glutamate/aspartate-binding site. It includes a contribution from carboxyl groups of D444, a residue that is essential for glutamate binding (Teichman & Kanner 2007). Because K^+^ binding to this site is predicted to be mutually exclusive with glutamate binding (tables 1 and 2), it is an interesting candidate for a K^+^ countertransport site.

In order to further identify glutamate–cation interactions and to quantify the reciprocal effects of cations on glutamate affinity, we docked glutamate into the EAAT3 homology model using the Gold docking program and estimated relative affinity using the ChemScore scoring algorithm (Ferrara *et al*. 2004). We also extracted the Δ*G* component of the ChemScore function in order to estimate the magnitude of energy change between the different conformational and cation-bound protein states. As expected from valence mapping, docking results indicate that Na^+^ positioned at site 3 increases the estimated affinity of glutamate for the EAAT3-binding site, as reflected by both the ChemScore and Δ*G* calculations (table 2). The docking results in conjunction with the valence estimates for site 3 in the glutamate-bound and apo forms suggest that Na^+^ bound at site 3 stabilizes bound glutamate in the correct orientation by interacting with the glutamate γ-carboxyl group.

## 6. Conclusions

A combination of crystallographic, kinetic and electrostatic modelling data is beginning to provide a framework for understanding the mechanisms underlying the coupled fluxes of glutamate and alkali cations in glutamate transporters. Electrostatic modelling is consistent with crystallographic data, indicating that binding site 2 for Na^+^, coordinated by dipoles formed from the α-helices in the HP2 loop and TM7, is favoured by the transporter state in which the substrate is occluded. The highly conserved NMDG motif that disrupts the α-helix in TM7 facilitates dipole interactions with Na^+^ and suggests a possible ubiquitous Na^+^ ion-binding site across all homologous transporters. Boudker *et al*. suggest that Na^+^ (Tl^+^) binding to this site may act to lock the HP2 loop into the occluded conformation. Our data help to substantiate this idea as well as indicate that glutamate must be bound and occluded in order for the closed HP2-loop conformation to be stable. This implies that Na^+^ binding at site 2 must occur concurrently with or following glutamate binding.

Analysis of the electrostatic mapping indicates a novel binding site for a third sodium ion that is not seen as a Tl^+^ density in the Glt_Ph_ crystal structure. This is consistent with the poor valence predicted for Tl^+^ at this site (table 1). Cooperative binding of Na^+^ and glutamate is a kinetic hallmark of the transporters. The γ-carboxyl group of glutamate is required for the stable coordination of Na^+^ at site 3, and occupancy of site 3 by Na^+^ has a reciprocal effect on glutamate affinity, suggesting a mechanism for this cooperativity. In a recent molecular dynamics study of Glt_Ph_, interactions between bound glutamate carboxyl groups and Na^+^ were found to stabilize the complex in the binding pocket, consistent with this notion (Shrivastava *et al*. 2008).

**Figure 4:**
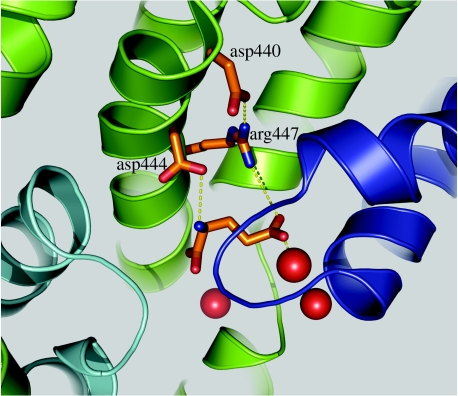
Potential binding pocket interactions facilitating coordinated substrate and Na^+^ binding (see §6).

Residue R447 in EAAT3 also interacts with the γ-carboxyl group of glutamate and is responsible for the recognition of acidic amino acid substrates (Bendahan *et al*. 2000; Yernool *et al*. 2004). Neutralization of an aspartate residue (D440) proximal to R447 in TM8 reduces both glutamate and Na^+^ apparent affinities (Tao & Grewer 2007). The effect of this mutation on Na^+^ binding was exclusive to the glutamate-bound but not the apo form of the transporter. Our electrostatic mapping of Na^+^ at site 3 could explain these findings through an interaction of D440 with R447 that positions R447 for glutamate binding (figure 4). Thus, neutralization of D440 would disrupt the orientation of R447, which, in turn, would disrupt the interaction of the transporter with the glutamate γ-carboxyl group. The binding of Na^+^ at site 3 would be reciprocally affected by reducing the likelihood that glutamate is situated for Na^+^ coordination.

The properties of the Na^+^ coordination sites identified by electrostatic mapping suggest that site 1 may be occupied before glutamate binding, while the sites 2 and 3 involve coordinated interactions with bound glutamate and the HP2 loop. The K^+^-selective binding site predicted here is coordinated in part by D444, which has been shown to affect substrate affinity in EAAT3 (Teichman & Kanner 2007) and is part of the aspartate-binding site in the Glt_Ph_ crystal structure (Boudker *et al*. 2007). The interaction of K^+^ at this site excludes binding of amino acid substrates (table 1; figure 3), and thus could provide a simple potential mechanism for K^+^ countertransport, another hallmark of glutamate transporter function (Kanner & Sharon 1978).

## Figures and Tables

**Table 1:** Electrostatic calculations for Na^+^- and K^+^-binding sites in EAAT3. (Sites 1–4 refer to potential cation-binding sites within 10 Å of the amino acid-binding site for the EAAT3 homology models and the Glt_Ph_ X-ray crystal structures. The EAAT3 and Glt_Ph_ models used for the valence calculations are in either the HP2-loop open or HP2-loop closed conformations and either the substrate-bound (glutamate or aspartate) or apo form.)

	site 1	site 2	site 3	site 4
EAAT3				
sodium				
EAAT3wt|open|apo	0.80	<0.3	0.72	0.84
EAAT3wt|open|l-glu	0.80	<0.3	0.72	<0.3
EAAT3wt|closed|apo	1.04	0.93	0.62	0.97
EAAT3wt|occluded|l-glu	1.04	0.93	0.91	<0.3
thallium				
EAAT3wt|open|apo	0.91	<0.3	<0.3	0.56
EAAT3wt|closed|apo	0.80	0.84	<0.3	0.94
EAAT3wt|occluded|l-glu	0.78	0.79	<0.3	<0.3
potassium				
EAAT3wt|open|apo	1.16	<0.3	<0.3	1.11
EAAT3wt|open|l-glu	1.16	<0.3	<0.3	<0.3
EAAT3wt|closed|apo	1.13	1.21	0.87	1.44
EAAT3wt|occluded|l-glu	1.13	1.21	<0.3	<0.3
caesium				
EAAT3wt|open|apo	1.03	<0.3	<0.3	0.90
EAAT3wt|closed|apo	0.71	0.83	<0.3	1.07
EAAT3wt|occluded|l-glu	0.71	0.83	<0.3	<0.3
Glt_Ph_				
sodium				
Glt_Ph_|open|apo	1.27	0.56	0.91	0.81
Glt_Ph_|open|l-asp	1.27	0.56	0.91	<0.3
Glt_Ph_|occluded|l-asp	1.05	1.05	0.61	<0.3
thallium				
Glt_Ph_|open|TBOA	1.09	0.46	<0.3	<0.3
Glt_Ph_|occluded|l-asp	1.07	1.08	<0.3	<0.3

**Table 2:** Substrate docking studies showing the effects of Na^+^ on binding affinity in the EAAT3 homology models and the Glt_Ph_ X-ray crystal structures. (The HP2-loop occluded or open models were docked with l-glutamate and l-aspartate using the Gold software program and the structures were scored using ChemScore, where higher ChemScore values reflect better fitness for docked poses. All models contained either no Na^+^ ions or Na^+^ ions at site 1 (Na1), sites 1 and 2 (Na1+Na2), sites 1 and 3 (Na1+Na3) or sites 1, 2 and 3 (Na1+Na2+Na3).)

	ChemScore	Δ*G* (kJ mol^−1^)
EAAT3|l-glu|no Na|occluded	13.17	−17.08
EAAT3|l-glu|Na1|occluded	14.36	−18.01
EAAT3|l-glu|Na1+Na2|occluded	14.11	−17.83
EAAT3|l-glu|Na1+Na3|occluded	17.61	−26.20
EAAT3|l-glu|Na1+Na2+Na3|occluded	18.45	−27.00
Glt_Ph_|l-glu|Na1+Na2|occluded	14.74	−23.54
Glt_Ph_|l-glu|Na1+Na2+Na3|occluded	18.42	−26.35
Glt_Ph_|l-asp|Na1+Na2|occluded	20.68	−24.00
Glt_Ph_|l-asp|Na1+Na2+Na3|occluded	23.71	−28.00
